# A Computational Drug Metabolite Detection Using the Stable Isotopic Mass-Shift Filtering with High Resolution Mass Spectrometry in Pioglitazone and Flurbiprofen

**DOI:** 10.3390/ijms141019716

**Published:** 2013-09-30

**Authors:** Masashi Uchida, Mitsuhiro Kanazawa, Atsushi Ogiwara, Hiroshi Sezaki, Akihiro Ando, Yohei Miyamoto

**Affiliations:** 1 Toxicology and Pharmacokinetics Laboratories, Pharmaceutical Research Laboratories, Toray Industries, Inc., 6-10-1 Tebiro, Kamakura, Kanagawa 248-8555, Japan; E-Mails: masashi_uchida@nts.toray.co.jp (M.U.); akihiro_ando@nts.toray.co.jp (A.A.); 2 Reifycs Inc., AIOS Toranomon 10F, 1-6-12 Nishishinbashi, Minato-ku, Tokyo 105-0003, Japan; E-Mails: kanazawa@reifycs.com (M.K.); ogiwr@reifycs.com (A.O.); 3 Measurement Assistance Center, Agilent Technologies Japan, Ltd., 9-1 Takakura, Hachioji, Tokyo 192-8510, Japan; E-Mail: email_japan@agilent.com

**Keywords:** drug metabolites, metabolites identification, mass spectrometry (MS), stable isotope, analytical software

## Abstract

The identification of metabolites in drug discovery is important. At present, radioisotopes and mass spectrometry are both widely used. However, rapid and comprehensive identification is still laborious and difficult. In this study, we developed new analytical software and employed a stable isotope as a tool to identify drug metabolites using mass spectrometry. A deuterium-labeled compound and non-labeled compound were both metabolized in human liver microsomes and analyzed by liquid chromatography/time-of-flight mass spectrometry (LC-TOF-MS). We computationally aligned two different MS data sets and filtered ions having a specific mass-shift equal to masses of labeled isotopes between those data using our own software. For pioglitazone and flurbiprofen, eight and four metabolites, respectively, were identified with calculations of mass and formulas and chemical structural fragmentation analysis. With high resolution MS, the approach became more accurate. The approach detected two unexpected metabolites in pioglitazone, *i.e*., the hydroxypropanamide form and the aldehyde hydrolysis form, which other approaches such as metabolite-biotransformation list matching and mass defect filtering could not detect. We demonstrated that the approach using computational alignment and stable isotopic mass-shift filtering has the ability to identify drug metabolites and is useful in drug discovery.

## 1. Introduction

Identifying metabolites is crucial to the drug discovery process. Generally, drug metabolism is a detoxification step *in vivo* [1]. In the last decade, assessing and understanding of drug metabolites has increased in importance. The U.S. Food and Drug Administration (FDA) established guidelines for testing the safety of metabolites [2]. Based on the nature of chemical reactions involved, metabolites formed from some Phase-I reactions are more likely to be chemically reactive or pharmacologically active and/or toxic, and therefore, more likely to need safety evaluation [2]. The guidelines also note that metabolic profiles can vary across species both quantitatively and qualitatively, hence early identification of human-specific metabolites and safety assessments based on the systemic exposure expected in humans are needed to develop new candidates progressively without delay. However, in many drug identification studies, the analytical signals derived from drug metabolites are weaker than the signals from biological matrices and analytical noise [3]. Therefore, it is difficult to identify these metabolites accurately and comprehensively in biological fluids. A radiolabeled compound is usually used to resolve these problems and identify metabolites due to its high sensitivity and specificity [4]. One can easily detect radiation as signals for the parent drug and its metabolites even if the metabolites’ signals are minor. However, radiolabeling has two major disadvantages; the risk of exposure in humans and a lack of information on the chemical structure of detected metabolites since the signals from the radiolabeled compound and its metabolites are the same.

In drug discovery, mass spectrometry (MS) combined with liquid chromatography (LC) is widely used to identify metabolites [4]. LC-MS has high sensitivity and selectivity and can rapidly reveal the mass and intensity of a target compound in biological fluids. Many types of MS and metabolite identification using MS have been developed. High resolution mass spectrometry including TOF-MS and Fourier transform mass spectrometry (FT-MS) can identify the masses of drug metabolites more accurately [5,6]. Tandem mass spectrometry (MS/MS) has great potential for chemical structural identification [7]. In addition, analytical software for drug metabolite identification makes it easier and faster to capture target compounds with typical analytical tools like metabolite biotransformation lists and/or mass defect filtering [8,9]; however, there are shortcomings associated with the use of MS and analytical software. In MS sensitivity and selectivity were both dependent on the physicochemical properties of target molecules, e.g., metabolites, and specificity may refer to the potential for background signals against the targets from unknown molecules, e.g., biological matrixes. Using analytical software, a general metabolite biotransformation list including major Phase-I and Phase-II metabolic pathways is not enough for comprehensive identification in many cases [8,9]. Furthermore, some metabolites have high mass defects and cannot be captured with the usual mass defect filtering [10,11]. While rapid and comprehensive drug identification is needed, there seems to be no completely satisfactory approach. We have developed a post-processing filtering approach for identifying drug metabolites using MS and new analytical software.

A stable isotope is a chemical isotope that is not radioactive. A stable isotope-labeled compound and its un-labeled parent have nearly the same chemical and physical characteristics, and behave almost identically [12–14]. In drug discovery, stable isotope-labeled compounds are widely used as internal standards for the quantification and as tracers for the structural identification of candidates and their metabolites [12–14]. In addition, loss or retention of isotope-labeled substituents at specific positions can afford valuable information on sites of metabolic attack in drug metabolism [13,14]. In drug metabolite identification, the isotope cluster technique is suitable for the metabolic profiling of drug substances. The “twin ions” derived from labeled and unlabeled compounds can facilitate the detection of unknown metabolites by mass spectral analysis [15]. Tang *et al*. identified unknown metabolites of amobarbital in urine using equimolar mixtures of [^15^N_2_] labeled and un-labeled compounds and comparing the spectra with gas chromatography [16]. When stable isotope-labeled and non-labeled compounds are biologically metabolized under the same conditions and analyzed by LC-MS individually, the MS data would indicate nearly the same response, but also show particular differences derived from the labeled isotope. Our results indicated that filtering a specific mass-shift equal to the labeled isotopic mass represents a fast and comprehensive method for identification of drug metabolites. In the present study, we used a stable isotope as a detection tool in drug metabolite identification, and have developed this approach with new analytical software that can visually filter particular ions having a specific mass-shift. A computationally accurate alignment of detected ions in LC/MS data enables the detection and visual inspection of twin ions, which are derived from different LC/MS measurements.

## 2. Results and Discussion

### 2.1. Metabolite Detection Using Isotopic Mass-Shift Filtering

**Figure 1:**
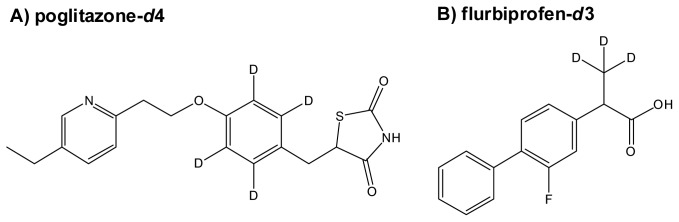
Chemical structures of pioglitazone-*d*_4_ (**A**) and flurbiporfen-*d*_3_ (**B**).

**Figure 2:**
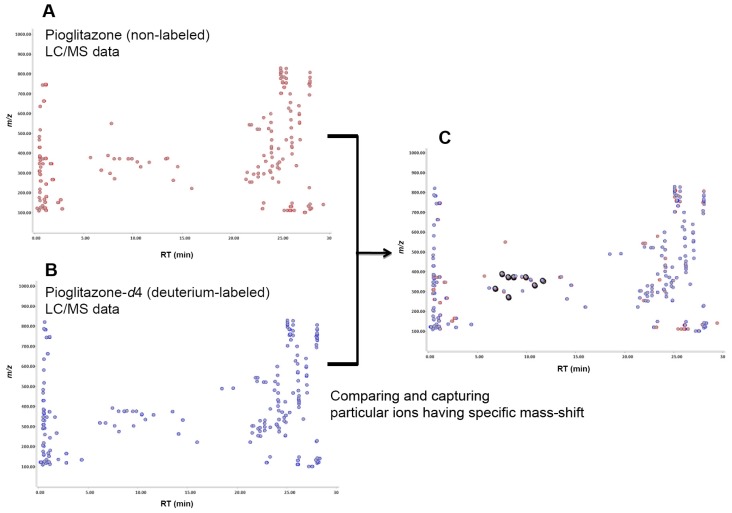
The concept of drug metabolite detection using stable isotopic mass-shift filtering. LC-MS data sets for un-labeled (**A**) and labeled (**B**) compounds were aligned, and the ions having a specific mass-shift equal to the mass of the labeled isotope were computationally filtered and visualized using the Signpost MS software (**C**). Metabolite ions, derived from the un-labeled compound and the labeled compound, are shown in red and blue, respectively.

**Figure 3:**
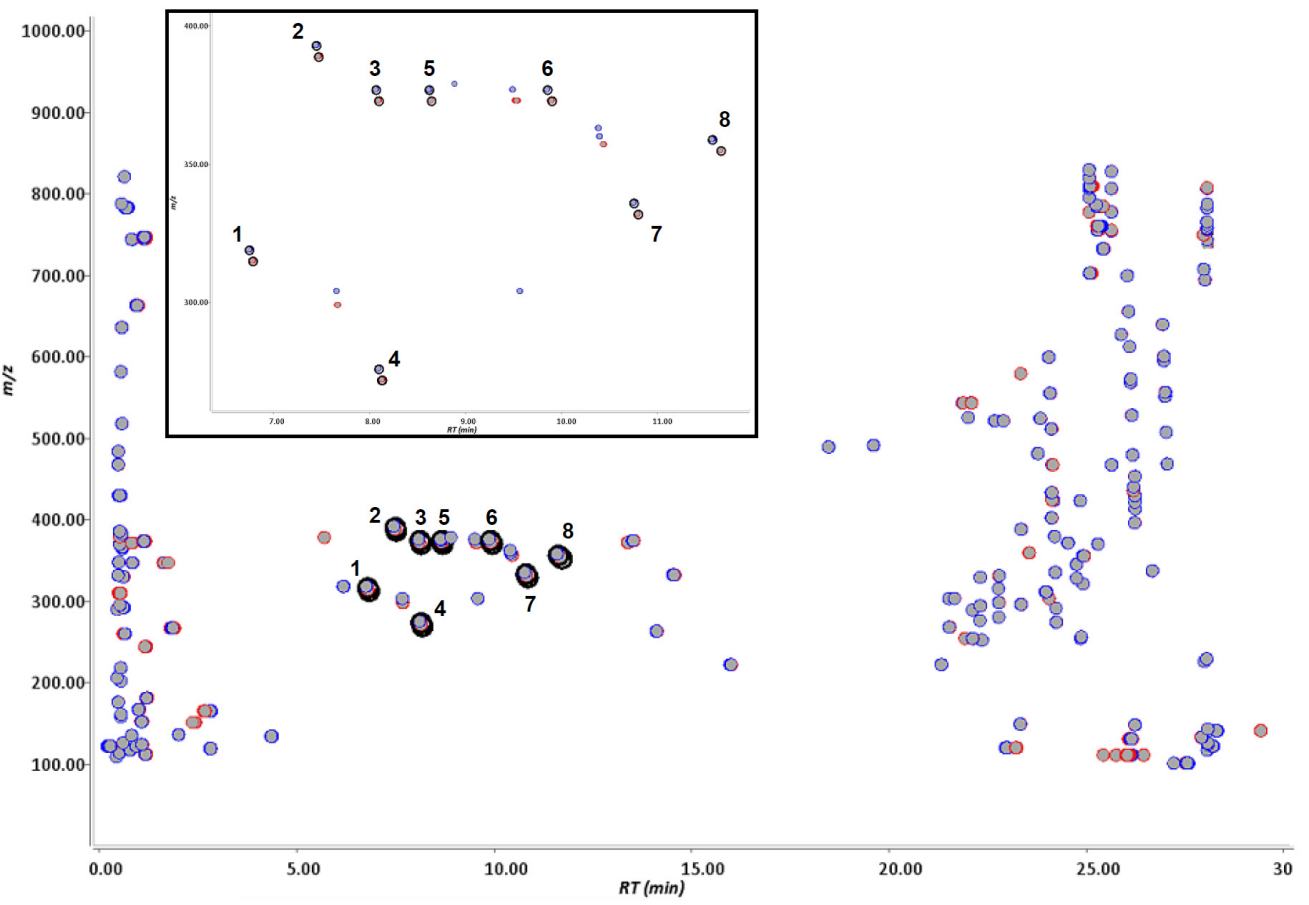
Ions detected by stable isotope mass-shift filtering between deuterium-labeled and un-labeled pioglitazone metabolized in human liver microsomes. The ions derived from un-labeled and labeled pioglitazone are shown by red and blue spots, respectively. By comparing the un-labeled (**red**) and labeled (**blue**) compounds, the particular ions having a specific mass-shift equal to 4 ± 0.04 Da were computationally filtered and visualized by being circled in black. Extended figures of captured ions are shown above.

**Figure 4:**
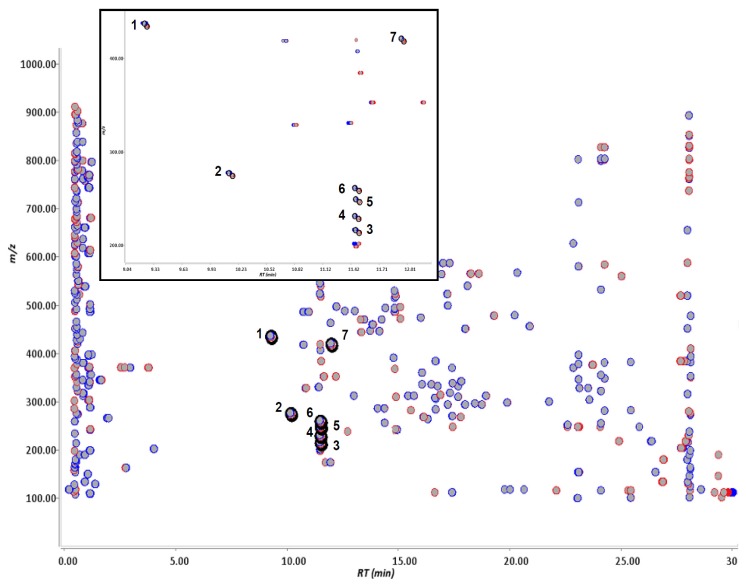
Ions detected by stable isotopic mass-shift filtering between deuterium-labeled and un-labeled flurbiprofen metabolized in human liver microsomes. The ions derived from un-labeled and labeled flurbiprofen were described in red and blue based on the mass spectrometric data, respectively. By comparing un-labeled (**red**) and labeled (**blue**) compounds, the particular ions having a specific mass-shift equal to 3 ± 0.03 Da were computationally filtered and visualized by being circled in black. Extended figures of captured ions are shown above.

**Figure 5:**
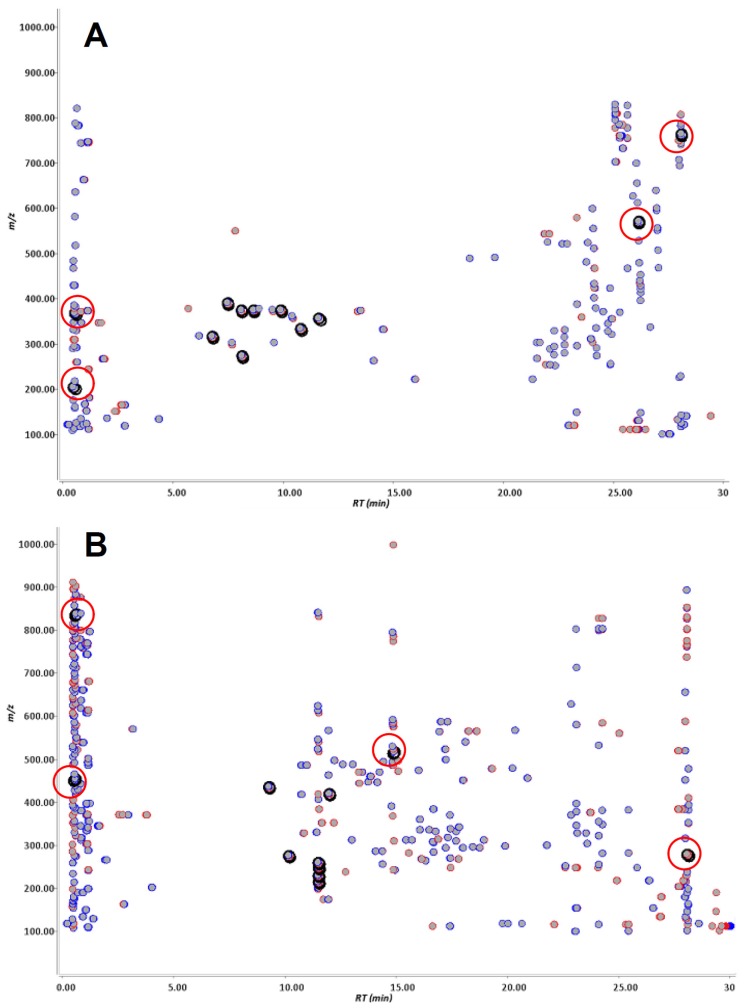
Ions detected by stable isotopic mass-shift filtering between deuterium-labeled and un-labeled compounds in pioglitazone (**A**) and flurbiprofen (**B**) metabolized in human liver microsomes. The ions derived from un-labeled and labeled compounds are shown in red and blue, respectively. By comparing un-labeled (**red**) and labeled (**blue**) compounds, the particular ions having a specific mass-shift equal to 4 ± 0.5 Da for pioglitazone and 3 ± 0.5 Da for flurbiprofen were computationally filtered and visualized by being circled in black. The ions, estimated as non-metabolites, were manually marked in red circle.

Pioglitazone, pioglitazone-*d*_4_, flurbiprofen and flurbiprofen-*d*_3_ were incubated in human liver microsomes, individually, and these reaction mixtures were analyzed by LC-TOF-MS separately. Pioglitazone-*d*_4_ and furbiprofen-*d*_3_ used were shown in Figure 1. The data were processed with Signpost MS software, which we developed. The data from the deuterium-labeled and un-labeled compounds were aligned, and then particular ions that had the same retention times, a similar signal intensity and also a specific mass-shift of 4.00 ± 0.04 Da and 3.00 ± 0.03 Da were captured in pioglitazone and flurbiprofen, respectively. The concept of detecting drug metabolites using mass-shift filtering is illustrated in Figure 2. In addition, the software also could process the data in a different manner in which the data were derived from a mixture of deuterated and non-deuterated compounds. Eight ion pairs were detected in pioglitazone and seven in flurbiprofen (Figures 3 and 4), as listed in Table 1. The ions were automatically filtered and visualized using the Signpost MS software (Figures 3 and 4). The retention times of deuterium-labeled ions were all shorter than those of un-labeled compounds. These differences due to the deuterium isotope are well known [16]. The signal intensity of the labeled compound was not constant per ion pair. Wang has reported the deuterium isotope’s effects in LC/MS and these effects affected the signal intensities of analytes due to a different degree of ion suppression between deuterium-labeled compound and its parent compound [17]. In the study, the deuterium effects of the LC/MS analyses were in a range, ±0.1 min for retention time and ±50% for signal intensity. In addition, we carried out low-resolution analyses and set a mass-shift to 4 ± 0.5 Da for pioglitazone and pioglitazone-*d*_4_, and 3 ± 0.5 Da for flurbiprofen and flurbiprofen-*d*_3_, and then detected four more ion pairs that were not metabolites but unknown signals from the matrix in both pioglitazone and flurbiprofen analyses (Figure 5). Furthermore, when signal intensity was also neglected in the mass-shift filtering, then four and ten more ion pairs, which originated from background noise were detected (data not shown).

### 2.2. Calculation of Mass and Estimation of Formula of Metabolites

For un-labeled compounds, each ion captured by the mass-shift filtering was processed to calculate its mass, and a formula was estimated from the observed mass using MassHunter software (Table 2). Based on these results, most of the formulas from pioglitazone were found to have one sulfur atom, and all ion formulas from flurbiprofen were found to have one fluorine atom. There were no significant differences between masses of estimated ion formula and observed masses in the ions detected in pioglitazone and flurbiprofen. Unfortunately, noise on the mass spectrum near *m/z* 214 interrupted calculation of the mass of *m/z* 214 in flurbiprofen. Four ions, *m/z* 259, 247, 229 and 214, derived from flurbiprofen had the same retention time, 11.50 min. We considered *m/z* 247 (259 –CH_2_), 229 (259 –CH_3_O) and 214 (259 –HCOOH) as all in-source fragmentation ions from their precursor ion, *m/z* 259, based on their retention times and ion formulas in flurbiprofen.

### 2.3. Structural Analysis of Metabolites

**Figure 6:**
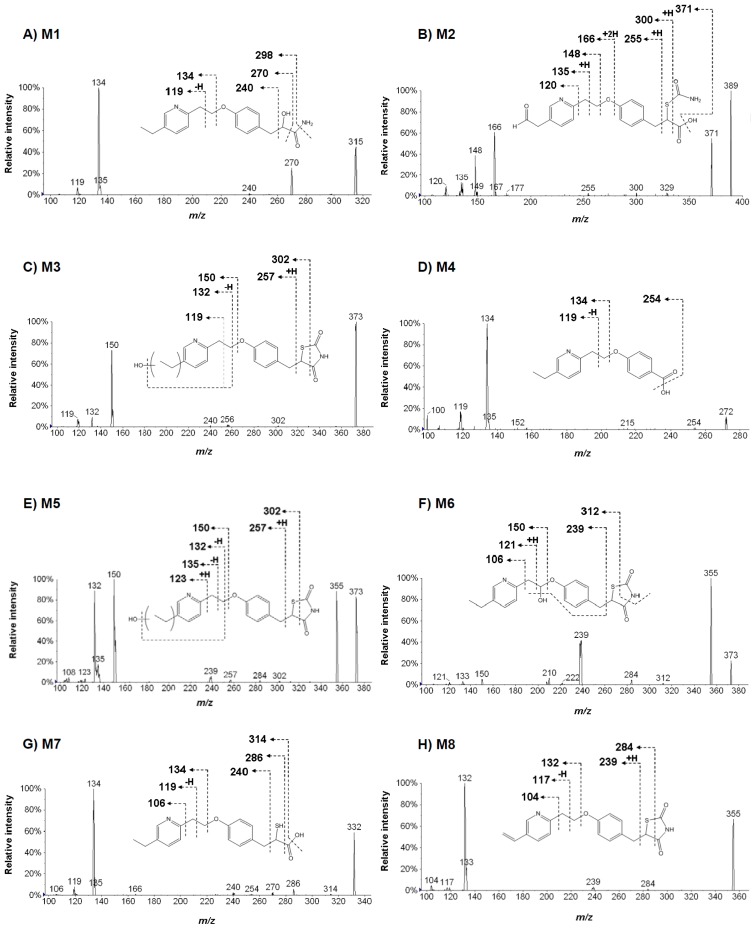
Representative MS/MS spectra of eight pioglitazone metabolites and their estimated chemical structures. Precursor ions were *m/z* 315 (**A**), 389 (**B**), 373 (**C**), 272 (**D**), 373 (**E**), 373 (**F**), 332 (**G**) and 355 (**H**) with the positive ion mode. Each arrow indicates a possible site of fragmentation, with the corresponding ion.

**Figure 7:**
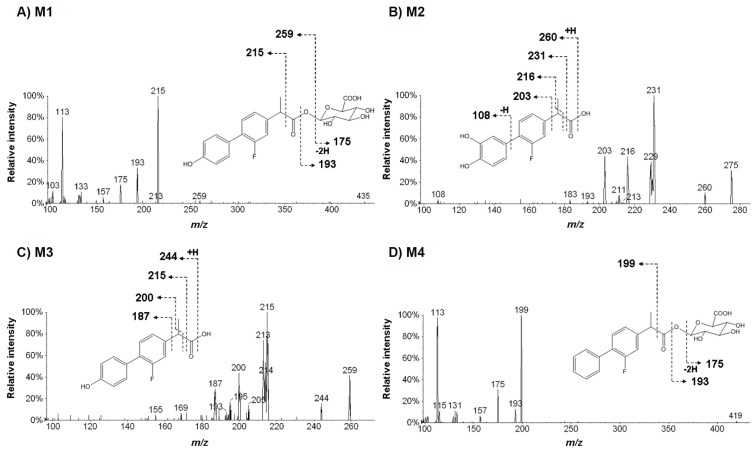
Representative MS/MS spectra of four flurbiprofen metabolites and their estimated chemical structures. Precursor ions were *m/z* 435 (**A**), 275 (**B**), 259 (**C**), and 419 (**D**) with the positive ion mode. Each arrow indicates a possible site of fragmentation, with the corresponding ion.

Structural analysis was carried out using a product ion scanning against ions regarded as metabolites by triple quadrupole mass spectrometry. In pioglitazone, precursor ions were set at *m/z* 315, 389, 373, 272, 332 and 355, and collisionally dissociated with nitrogen gas being set at 30 eV. In flurbiprofen, precursor ions were set at *m/z* 435, 275, 259 and 419, and dissociated in the same way as pioglitazone. Product ion spectra and estimated chemical structures of these precursors are shown in Figures 6 and 7. In pioglitazone, major product ions from the dissociation of *O*-alkyl chain in the middle of its structure were found in all of the metabolites (Figure 6). In flurbiprofen, typical product ions from the dissociation of acyl-glucuronide were found in M1 and M4 (Figure 7A,D). Based on these results, eight and four metabolites were proposed in pioglitazone and flurbiprofen, respectively. Additionally, values of mass defects from parent were calculated for each metabolite. These results are summarized in Table 3. With two other approaches, a metabolite-biotransformation list matching and a mass defect filtering, we could not find any other metabolite except those found by the mass-shift filtering in the study. The mass-shift detection captured several unexpected metabolites, M1 and M2 in pioglitazone, which the two other approaches did not detect. A metabolite-biotransformation list matching and a mass defect filtering are both widely used and facilitate metabolite identifications in drug discovery [8,9]. However, usability of the metabolite-biotransformation list is sometimes limited because of the unexpected metabolic pathways of drug candidates [8,9]. Mass defect filtering sometimes also led to lost metabolites because some have unexpected high mass defects from their parents [11]. In pioglitazone, M1, the hydroxypropanamide form, and M2, the aldehyde hydrolysis form were both considered to be metabolites and also have relatively high mass defects, 43.6 and −10.2 mDa. The results of the study showed that the approach using the stable isotopic mass-shift filtering directly detects signals derived from labeled isotopes and is not influenced by the metabolic complexity. In this study, we also confirmed no existence of particular metabolites that lost the labeled site via chemical and biological desorption at the phenyl group of pioglitazone-*d*_4_ and the methyl group of furbiprofen-*d*_3_ using the software by other mass-shift parameters; 3 and 2 Da for pioglitazone, and 1 and 2 Da for flurbiprofen. It would be more suitable to label the parent drug at the core frame of its structure with stable isotopes, e.g., carbon-13, to avoid loss of labels during metabolism [18].

## 3. Experimental Section

### 3.1. Chemical and Reagents

Pooled human liver microsomes (mix gender) were purchased from Xenotech (Lenexa, KS, USA). Pioglitazone, pioglitazone-*d*_4_ (5-[[2,3,5,6-tetradeuterio-4-[2-(5-ethylpyridin-2-yl)ethoxy]phenyl]methyl]-1,3-thiazolidine-2,4-dione), flurbiprofen and flurbiprofen-*d*_3_ (2-Fluoro-α-trideuteromethyl-[1,1biphenyl]-4-acetic Acid) were purchased from TLC PharmaChem (Vaughan, ON, Canada). Human blank plasma was prepared from blood obtained from healthy volunteers in our facility. The experiments were conducted according to the ethical guideline for biological Experiments, Research and Development Division, Toray Industries, Inc.

### 3.2. *In Vitro* Metabolism Using Liver Microsomes

Pioglitazone, pioglitazone-*d*_4_, flurbiprofen and flurbiprofen-*d*_3_ (all 10 μmol/L) were incubated separately with pooled human liver microsomes (0.5 mg proten/mL), NADPH (1 mmol/L), and UDPGA (5 mmol/L) in 100 mmol/L sodium phosphate buffer (pH 7.4) for 60 min. The enzymatic reactions were initiated by the addition of an NADPH and UDPGA mixture. After a 60-min incubation, 10 μL of reaction mixture was added to 10 times the volume of human blank plasma (100 μL), and then each reaction was stopped by the addition of 3 times the volume of a methanol/acetonitrile mixture (1:1) (300 μL). The suspensions were centrifuged at 12,000 rpm for 10 min. Aliquots of the supernatants were analyzed by LC-TOF-MS and LC-MS/MS, respectively.

### 3.3. Liquid Chromatography

For each analysis of metabolites of pioglitazone, pioglitazone-*d*_4_, flurbiprofen and flurbiprofen-*d*_3_, HPLC was performed using Cadenza CD-C18 (2.0 × 100 mm, 3 μm, Imtakt, Tokyo, Japan). A gradient with buffer A (0.1% *v*/*v* formic acid) and buffer B (acetonitrile containing 0.1% *v*/*v* formic acid) was used at a flow rate of 0.6 mL/min. Buffer B was maintained at 1% for 1 min and then linearly increased to 60% in 20 min. The analytical column was washed with 99% B for 5 min and equilibrated with 1% B for 4 min before the next injection.

### 3.4. Time of Flight Mass Spectrometry

Mass spectra were recorded over the range *m/z* 100 to 1000 in pioglitazone, pioglitazone-*d*_4_, flurbiprofen and flurbiprofen-*d*_3_, and the analyses were carried out using a 6230 Accurate-Mass Time-of-Flight (TOF) MS system (Agilent Technologies, Santa Clara, CA, USA). The mass spectrometer was operated in the positive ion electrospray mode for pioglitazone and the negative mode for flurbiprofen with a capillary voltage of 3500 V and −3500 V, and drying gas temperature of 350 °C. Each of 4 compounds was also measured under the same conditions, to subtract compounds specific ions in the data analysis process later on. The recorded data was processed with MassHunter^®^ software ver.B.04.00 (Agilent technologies) to calculate the masses of the detected metabolites. The mass axis was calibrated using standard ESI tuning solution (Agilent Technologies) that was continuously sprayed into the ion source during each analysis. The reference masses were set at *m/z* 121.0509 and 922.0098.

### 3.5. Triple Quadrupole Mass Spectrometry

Product ion analyses of pioglitazone, flurbiprofen and their metabolites were carried out using the QTRAP 5500 MS/MS System (AB SCIEX, Framingham, MA, USA). The mass spectrometer was operated in the positive ion electrospray mode for pioglitazone and the negative mode for flurbiprofen with a capillary voltage of 5500 V and −4500 V, and source temperature of 650 °C. The MS/MS analysis was performed using nitrogen as the collision gas with the collision energy being set at 30 eV.

### 3.6. Alignment and Filtering of Stable Isotope Mass-Shifts between Labeled and Un-Labeled Compound Data Sets

The mass spectral data of the *in vitro* metabolic reaction mixture from the deuterium-labeled and un-labeled compounds, collected by separate LC-TOF-MS measurements, were processed with new Signpost MS software ver.1.2 (Reifycs, Tokyo, Japan) that we developed. The basic function of this software is to provide detection and alignment of ion peaks in multiple measurements of LC-MS. The detected and aligned data are presented visually in a spectrogram, a chromatogram, and a map view represented in a retention time-*m/z* plane. A computationally accurate alignment of detected ions in LC/MS data enables comparison of the two LC/MS measurements. We also expanded the visual presentation functions of the software to display and filter a stable isotope mass-shift.

The stable isotope mass-shift filtering accepts aligned LC/MS data of both labeled and un-labeled compounds. In the retention time-*m/z* map view, ions derived from labeled and un-labeled samples are shown in different colors (Figure 2A,B). Twin ions, which satisfy the conditions under which both labeled and un-labeled derived ions exist, have a specified mass-shift and similar signal intensity within the specified retention time range, and then are automatically extracted (Figure 2C). This function is used to discover metabolite candidates of the given compound, because such metabolites are expected to exist almost equally in both labeled and un-labeled metabolic reaction mixtures.

In the study, the following parameters were adopted to select twin ions in the stable isotope mass-shift filtering: Retention time tolerance, ±0.1 min and the drift of signal intensity, ±50%. We set a mass shift of 4.00 ± 0.04 Da for pioglitazone and pioglitazone-*d*_4_, and 3.00 ± 0.03 Da for flurbiprofen and flurbiprofen-*d*_3_.

## 4. Conclusions

In summary, we developed new software, Signpost MS, and demonstrated that an approach using the stable isotopic mass-shift filtering has the ability to identify drug metabolites and is very useful in drug discovery. We employed pioglitazone and flurbiprofen as model compounds and detected two unexpected metabolites in pioglitazone, *i.e*., the hydroxypropanamide form and the aldehyde hydrolysis form, which other approaches such as metabolite-biotransformation list matching and mass defect filtering could not detect. With high resolution MS, the approach became more accurate to filter a specific mass-shift. There was no biological desorption of the labeled site in this study. A deuterium-labeled compound is widely used and readily available, however it would be more suitable to label its core frame, *i.e*., a carbon by carbon-13, of the chemical structure. We demonstrated that the approach using computational alignment and stable isotopic mass-shift filtering has the ability to identify drug metabolites and is useful in drug discovery.

## Figures and Tables

**Table 1:** Ions detected by stable isotope mass-shift filtering between deuterium-labeled and un-labeled compounds in pioglitazone and flurbiprofen metabolized in human liver microsomes.

Parent compound (a)	Number of ion pairs	Retention time (min)	Δ Retention time (min) (b)	Intensity (%) (c)	Δ intensity (%) (d)	[M + H]^+/^/[M − H]^−^*m/z* (amu) (e)	Mass-shift Δ *m/z* (amu) (f)
Pioglitazone Pioglitazone-*d*_4_	1	6.78		1.71		315.17	
		6.74	−0.04	1.74	2	319.19	+4.02
	2	7.46		10.83		389.11	
		7.44	−0.02	6.59	−39	393.14	+4.03
	3	8.09		15.21		373.12	
		8.06	−0.03	13.65	−10	377.14	+4.02
	4	8.12		2.01		272.13	
		8.09	−0.03	1.89	−6	276.15	+4.02
	5	8.64		100.00		373.12	
		8.61	−0.03	67.07	−33	377.14	+4.02
	6	9.89		11.96		373.12	
		9.85	−0.04	7.07	−41	377.14	+4.02
	7	10.79		5.05		332.13	
		10.75	−0.04	4.66	−8	336.15	+4.02
	8	11.66		1.35		355.11	
		11.57	−0.09	1.28	−5	359.13	+4.02

Flurbiprofen Flurbiprofen-*d*_3_	1	9.26		4.28		435.10	
		9.24	−0.02	4.12	−4	438.12	+3.02
	2	10.16		5.30		275.07	
		10.13	−0.03	4.94	−7	278.08	+3.01
	3	11.50		59.97		214.08	
		11.46	−0.04	64.26	7	217.09	+3.01
	4	11.49		20.50		229.06	
		11.45	−0.04	18.76	−8	232.08	+3.02
	5	11.50		1.96		247.07	
		11.46	−0.04	2.21	13	250.09	+3.02
	6	11.50		7.17		259.07	
		11.45	−0.05	7.01	−2	262.09	+3.02
	7	11.97		94.39		419.11	
		11.94	−0.03	99.25	5	422.12	+3.01

(a) Above and below data are from un-labeled and deuterium-labeled compounds in each ion pair, respectively;

(b) Δ retention time = (retention time of deuterium-labeled compound) − (retention time of un-labeled compound);

(c) Relative signal intensity against the highest peak in each run. The highest peak is 100%;

(d) Δ intensity = [(deuterium-labeled compound − un-labeled compound)/un-labeled compound] × 100;

(e) Observed mass; [M + H]^+^: piogliazone/pioglitazone-*d*_4_; [M − H]^−^: flurbiprofen/flurbiprofen-*d*_3_;

(f) Δ *m/z* = (*m/z* of deuterium-labeled compound) − (*m/z* of un-labeled compound).

**Table 2:** Observed masses and estimated formula of ions detected by stable isotope mass-shift filtering in pioglitazone and flurbiprofen metabolized in human liver microsomes.

Parent compound	Number of ions	Retention time (min)	Observed accurate mass *m/z* (amu) (a)	Estimated ion formula (b) (mass, amu) (c)	Diff (ppm) (d)
Pioglitazone	1	6.78	315.17012	C_18_H_23_N_2_O_3_ (315.17032)	0.63
	2	7.46	389.11569	C_19_H_21_N_2_O_5_S (389.11657)	2.26
	3	8.09	373.12111	C_19_H_21_N_2_O_4_S (373.12165)	1.46
	4	8.12	272.12734	C_16_H_18_NO_3_ (272.12812)	2.09
	5	8.64	373.12123	C_19_H_21_N_2_O_4_S (373.12165)	1.14
	6	9.89	373. 12106	C_19_H_21_N_2_O_4_S (373.12165)	1.49
	7	10.79	332.13076	C_18_H_22_NO_3_S (332.13149)	2.22
	8	11.66	355.11028	C_22_H_15_N_2_O_3_ (355.11109)	2.28

Flurbiprofen	1	9.26	435.10972	C_21_H_20_FO_9_ (435.10968)	−0.07
	2	10.16	275.07257	C_15_H_12_FO_4_ (275.07251)	−0.21
	3	11.50	214.07995	N.E. (e)	-
	4	11.50	229.06721	C_14_H_11_FO_2_ (229.06703)	−0.79
	5	11.50	247.07723	C_14_H_13_FO_2_ (247.0776)	1.45
	6	11.50	259.07753	C_15_H_12_FO_3_ (259.07760)	0.26
	7	11.97	419.11524	C_21_H_20_FO_8_ (419.11477)	−1.11

(a) [M + H]^+^: piogliazone, [M − H]^−^: flurbiprofen;

(b) Estimated ion formula from observed mass;

(c) Mass of estimated ion formula;

(d) Difference of mass = (mass of estimated ion formula – observed mass)/observed mass × 10^6^;

(e) Not estimated because of low signal intensity and high background noise.

**Table 3:** Estimated *in vitro* human metabolites of pioglitazone and flurbiprofen using stable isotope mass-shift filtering.

Parent compound	Metabolite ID	Estimated formula (a) (mass, amu) (b)	Observed major production	Estimated metabolite form (c)	Mass defect (mDa) (d)
Pioglitazone (PIO)	M1	C_18_H_22_N_2_O_3_ (314.163043)	298, 270, 240, 134, 119	PIO hydroxypropanamide	43.6
	M2	C_19_H_20_N_2_O_5_S (388.109295)	371, 300, 255, 166, 148, 135, 120	Aldehyde PIO hydrolysis	−10.2
	M3	C_19_H_20_N_2_O_4_S (372.114380)	302, 256, 150, 132, 119	Hydroxy PIO	−5.1
	M4	C_16_H_17_NO_3_ (271.120844)	254, 134, 119	PIO benzoic acid	1.4
	M5	C_19_H_20_N_2_O_4_S (372.114380)	302, 257, 150, 135, 132, 123	Hydroxy PIO	−5.1
	M6	C_19_H_20_N_2_O_4_S (372.114380)	312, 239, 150, 121, 106	Hydroxy PIO	−5.1
	M7	C_18_H_21_NO_3_S (331.124216)	314, 286, 240, 134, 119, 106	PIO mercaptopropanoic acid	4.8
	M8	C_22_H_14_N_2_O_3_ (354.100443)	284, 239, 132, 117, 104	Dehydro PIO	−19.0
	parent	C_19_H_20_N_2_O_3_S (356.119465)	- (e)	PIO	-

Flurbiprofen (FBP)	M1	C_21_H_21_FO_9_ (436.116963)	259, 215, 193, 175	Hydroxyl FBP glucuronide	27.0
	M2	C_15_H_13_FO_4_ (276.079788)	260, 231, 216, 203, 108	Di-hydroxy FBP	−10.2
	M3	C_15_H_13_FO_3_ (260.084873)	244, 215, 200, 187	Hydroxy FBP	−5.1
	M4	C_21_H_21_FO_8_ (420.122048)	199, 195, 175	FBP glucuronide	32.1
	parent	C_15_H_13_FO_2_ (244.089958)	- (e)	FBP	-

(a) Estimated formula; pioglitazone: Estimated ion formula −H, flurubiprofen: Estimated ion formula +H;

(b) Mass of estimated formula;

(c) Estimated chemical structures of metabolite form estimated formula and observed fragment ions;

(d) Mass defect from parent = (mass of estimated metabolite after the decimal point – mass of parent after the decimal point) × 10^3^;

(e) Not conducted.
